# Changes in proportional mortality from diabetes and circulatory disease in Mauritius and Fiji: possible effects of coding and certification

**DOI:** 10.1186/s12889-019-6748-7

**Published:** 2019-05-02

**Authors:** Stephen Morrell, Richard Taylor, Devina Nand, Chalapati Rao

**Affiliations:** 1 School of Public Health and Community Medicine (SPHCM), Faculty of Medicine University of New South Wales (UNSW), Sydney, Australia; 2 Fiji Ministry of Health, Suva, Fiji; 3 Department of Global Health, Research School of Population Health, Australian National University, Canberra, Australia

**Keywords:** Diabetes, Mortality, Cardiovascular disease, Trends, ICD coding

## Abstract

**Background:**

Many developing countries are experiencing the epidemiological transition, with the majority of deaths attributed to cardiovascular disease, cancer, Type 2 diabetes (T2DM) and others. In some countries, large proportional mortality attributed to diabetes is evident in official mortality statistics, with Mauritius and Fiji rated as the highest in the world.

**Methods:**

This study investigates trends in recorded diabetes and cardiovascular disease mortality in Mauritius and Fiji under coding from the International Classification of Diseases (ICD) versions 9 and 10, using mortality data reported from these countries to the World Health Organization (WHO).

**Results:**

In Mauritius over 1981–2004, T2DM proportional mortality varied between 4% and 7% in males (M) and 5% and 9% in females (F). In 2005 there was a sudden increase to M 20% and F 25%, which continued to M 25% and F 30% by 2012. Over 1981–2004 the proportion of circulatory disease mortality rose from 44% to 49% in males, and from 46% to 57% in females. In 2005, circulatory disease mortality proportions fell precipitously to 34% in males and 37% in females, and declined to 31% and 34% by 2013. ICD–10 coding was introduced in 2005.

In Fiji, sharp rises in proportional T2DM mortality from 3% in both sexes in 2001 to M 15% and F 20% in 2002 were followed by more gradual trend increases to M 20% and F 26% by 2012–13. Circulatory disease proportions fell steeply from M 57% and F 53% in 2001 to M 44% and M 38% by 2004, with subsequent less steep declines to M 39% and F 30% by 2012. ICD–10 coding was introduced in 2001.

**Conclusions:**

Large, abrupt changes in diabetes and circulatory disease proportional mortality in Fiji and Mauritius coincided with the local introduction of ICD–10 coding in different years. There is also evidence for diabetes-related misclassification of underlying cause of death in Australia and the USA. These artefacts can undermine accurate monitoring of cause of death for evaluation of effectiveness of prevention and control, especially of circulatory disease mortality which is demonstrably reversible in populations.

## Background

Accurate magnitude and secular trends of mortality by cause are important for monitoring population effects of possible causative influences, and effectiveness of prevention and control measures. Various international sources indicate considerable variability in rates and proportions of diabetes mortality derived from official country mortality statistics. The WHO reports diabetes mortality for 2012 (age standardised, both sexes) as 175/10 ^5^for Mauritius, [1] close to the estimate of 174/10^5^ shown on the World Life Expectancy website [2]. For Fiji, WHO indicates diabetes mortality as 160/10^5^, [1] and the World Life Expectancy website estimates it as 147/10^5^. Mauritius and Fiji are ranked by the World Life Expectancy website as having the two highest diabetes mortality rates in the world [2]. The Institute for Health Metrics and Evaluation Global Burden of Disease (IHME GBD) estimates for 2015 indicate that diabetes is ranked first or second in years of life lost (YLL) in Mauritius and Fiji, depending on the combination or disaggregation of categories of cardiovascular disease.

A number of ‘bridge-coding’ studies have estimated changes in cause-specific mortality trends attributable to changeovers from ICD–9 to ICD–10 cause-of-death coding, including in the US, [3] and Europe [4–7]. The US study attributed a < 1% rise in mortality from diabetes complications to the coding changeover, [3] while a European study reported no significant changes in Norway, Sweden and Finland, but a 20% diabetes mortality increase in the Netherlands [4]. For cerebrovascular disease, the US study found a 6% increase attributable mostly to the ICD–10 rule change in classifying pneumonia (frequently an immediate terminal condition) as cerebrovascular disease if both are present on the death certificate; and a 2% decline in ‘heart conditions’ (ICD–10 codes I00–I09, I11, I13, I20–I51) due mainly to cardiac arrest being non-specific (and also likely immediately terminal), and classified as diabetes if diabetes is also present on the death certificate. While artefactual changes of these magnitudes would be expected to accompany changes to coding systems and rules, assessment of mortality changes accompanying coding changes may not be occurring in less developed countries. Despite extensive literature searches, the authors were not able to locate studies of mortality trends in developing countries in relation to cause-of-death coding changes.

Diabetes can result in specific diabetes-related complications, especially metabolic abnormalities (ketoacidosis, hyperosmolar states), renal disease and insufficiency, gangrene of the extremities, retinopathy and blindness, neuropathy, and others, as listed in successive editions of the ICD coding system, [8] several of which can lead directly to death. Along with blood pressure, blood lipid levels, and tobacco smoking, diabetes is also a risk factor for atherosclerotic cardiovascular disease which is a major cause of death in diabetics. Death from atherosclerotic coronary or cerebrovascular disease, with or without diabetes as a risk factor, is classified under circulatory disease as the underlying cause of death. There are no ICD codes under diabetes for these conditions. Diabetes without fatal diabetes-specific complications does not directly cause death, but can contribute to death, especially from cardiovascular disease. When a contributing risk factor for death, uncomplicated diabetes should be placed in Part II of the death certificate, the section reserved for contributing risk factors for death from a given cause.

Tabulation of publicly available WHO mortality data supplied by countries indicates a relatively high proportion of deaths attributable to diabetes in Mauritius and Fiji compared to other countries according to the latest available data [9]. We hypothesised that these proportions may be artefactual and possibly related to mortality coding changes, from ICD–9 to ICD–10. In the present study, secular trends in diabetes deaths are compared to trends in cardiovascular disease mortality in Mauritius and Fiji, and in relation to ICD versions used for derivation and coding of underlying cause of death. Secular trends in the same causes of death in Australia are provided for comparison.

## Methods

### Data sources

WHO mortality data were analysed by trends in proportional contributions of diabetes as underlying cause to total deaths using ICD–9 and ICD–10 codes for diabetes from ICD–9 Chapter 3 (‘Endocrine, Nutritional and Metabolic Diseases, and Immunity Disorders’) and ICD–10 Chapter IV (‘Endocrine, nutritional and metabolic diseases’). Contributions of circulatory disease were assessed from ICD–9 Chapter 7 and ICD–10 Chapter IX, ‘Diseases of the Circulatory System’, as underlying cause of death to overall (all-cause) mortality. These data are available by single year, sex and 5–year age group, with the corresponding ICD coding version varying across countries [10]. WHO mortality supplied by individual countries are coded according to the edition of ICD in use at the time of mortality registration in each country. These data are freely and publicly available at the WHO website [10].

For Mauritius, WHO mortality data spans 1981–2016. For Fiji, WHO annual death data from 2001 to 2012 are analysed. Although 1999 Fiji data are available from WHO for Fiji, 2000 data are missing, and there are significant and implausible differences in cause structure between 1999 and 2001, so 1999 data are not included in analyses. Mortality data were accessed from the most recently updated WHO mortality data available in April 2018. Corresponding data for Australia covering 1980–2015 are also plotted for comparison.

### Study design and analysis

Differences in population trends in proportions of diabetes and circulatory disease as underlying cause of death of total (all cause) mortality are plotted over time in relation to changes from ICD–9 to ICD–10 mortality coding. SAS version 9.4 was used for data extraction, calculation of proportions and exact binomial 95% confidence intervals, and for plotting trends.

## Results

**Fig. 1:**
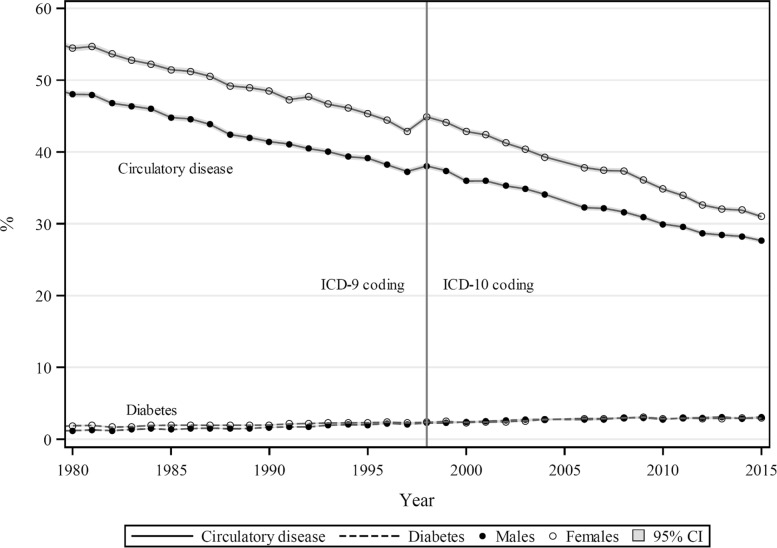
Proportions of total mortality (%) from diabetes and circulatory disease, by sex, Australia 1980–2015

Proportional circulatory disease mortality over 1980–2015 in Australia has shown steady declines, from 57% in males and 49% in females in 1980, to 31% and 28%, respectively by 2015 (Fig. 1). Diabetes proportional mortality has increased steadily over the corresponding period, from 2% to 4% of all mortality. No significant interruption to these trends is associated with the 1998 changeover from ICD–9 to ICD–10 coded mortality as reported to WHO.

**Fig. 2:**
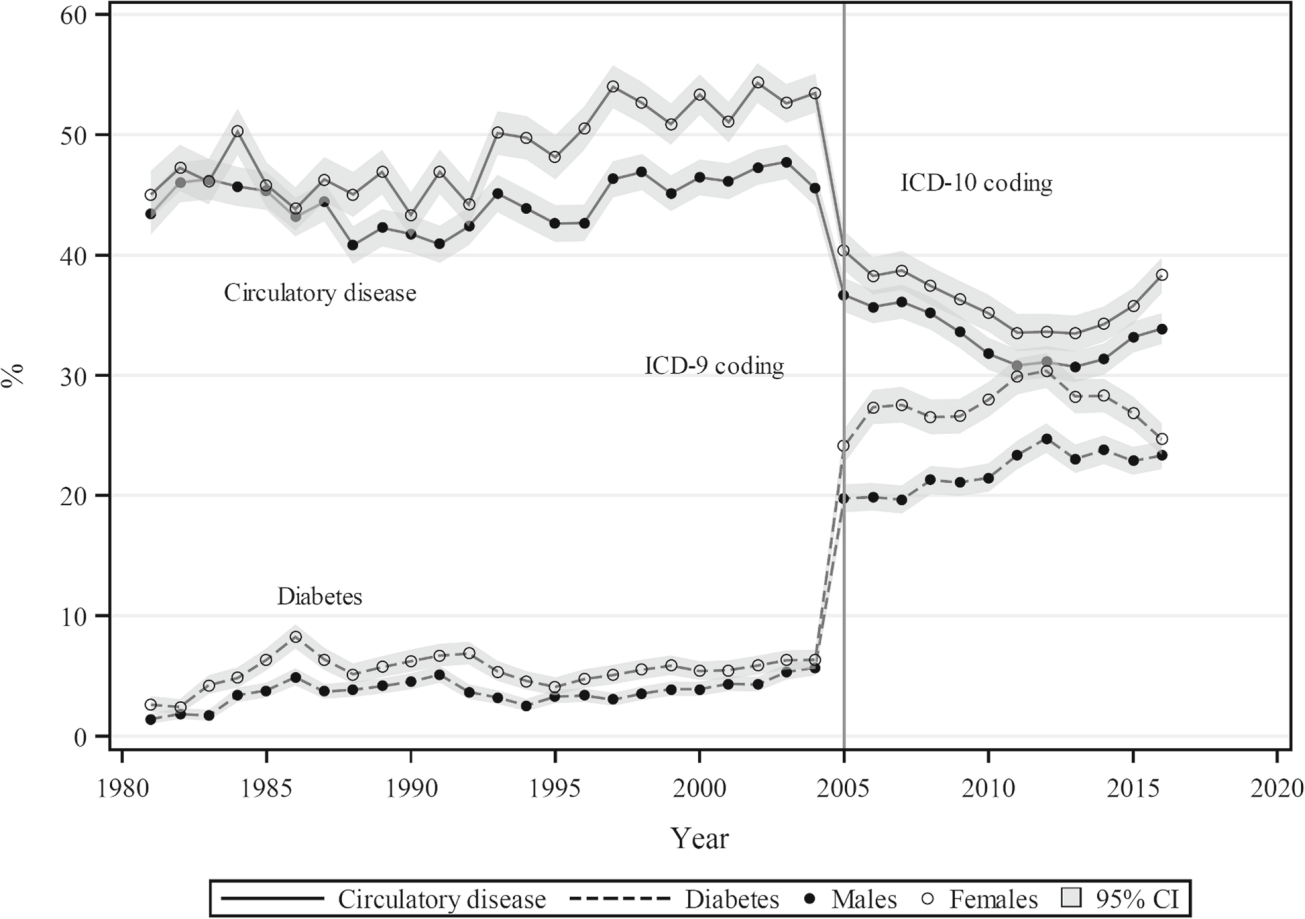
Proportions of total mortality (%) from diabetes and circulatory disease, by sex, Mauritius 1981–2016

In Mauritius over 1981–2004, diabetes proportional mortality showed no substantial trend, varying between 2–7% for males, and 3–9% in females. In 2005 there was a sudden increase to 20% in males and 25% in females, which increased further to 25% and 30% respectively by 2013 (Fig. 2). These represent absolute increases of 18–23% points or 3 to 5-fold to increases. Over 1981–2004 the proportion of circulatory disease mortality rose from 44% to 48% in males, and from 45% to 54% in females. In 2005, circulatory disease mortality proportions fell precipitously to 37% for males and 40% for females, followed by slower declines to 31% (males) and 34% (females) by 2013. These declines represented absolute decreases of 11–14% points, and proportionate declines of 24% and 26%, respectively, over 2004–2005. ICD–10 coding was introduced in Mauritius in 2005.

**Fig. 3:**
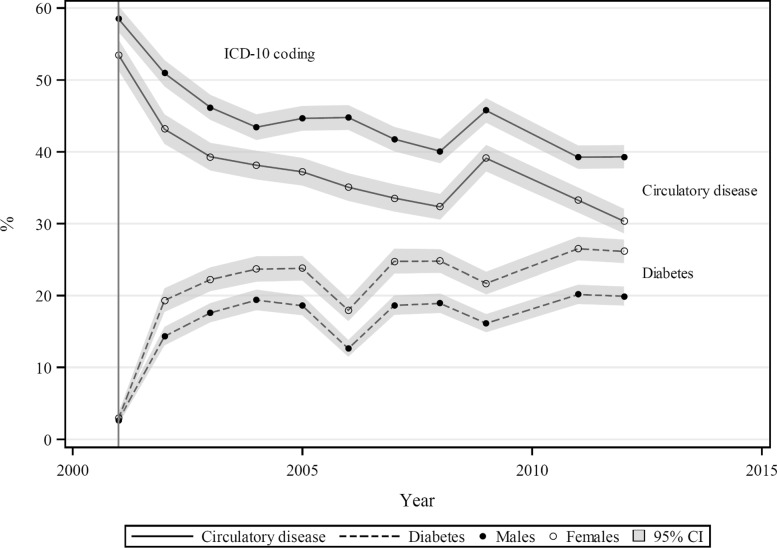
Proportions of total mortality (%) from diabetes and circulatory disease, by sex, Fiji 2001–2012

In Fiji, sharp rises in proportional diabetes mortality from 3% in both sexes in 2001 to 15% in males and 20% in females 2002 were followed by more gradual trend increases to 20% (males) and 27% (females) by 2012–13 (Fig. 3). These represented absolute increases of 17–25% points or 6 to 9-fold increases. Circulatory disease proportions fell steeply from 58% in males and 53% in females in 2001 to 44% and 38% respectively by 2004, with subsequent more gradual declines to 39% and 30% respectively by 2012–13. The absolute decrease was 19–23% points, representing corresponding 33–43% proportionate declines. WHO mortality data was coded according to ICD–10 in 2001, but effects attributable to the coding changeover did not manifest until 2002.

Averaged for both sexes, in both Mauritius and Fiji following the cause-of-death coding change, proportional mortality from diabetes increased from around 5% to 25% (five-fold increase of 20% points), and circulatory disease mortality declined by approximately 20% points from around 55% to 35% (36% proportional decline).

## Discussion

The World Life Expectancy website indicates that Mauritius and Fiji have the highest death rates from diabetes in the world, and in July 2016 an article appeared in the *Fiji Times Online* reporting that Fiji had the second highest diabetes mortality rate in the world, exceeded only by Mauritius [11].

The present study has taken a closer look at diabetes and circulatory disease proportional mortality trends in the two countries, from publicly available cause-specific mortality data accessible from WHO, [10] and has shown that these high levels appear to have stemmed from changes in cause-of-death coding practices. Three to five-fold increases in diabetes proportional mortality occurred in Mauritius concurrently with the change from ICD–9 to ICD–10 cause-of-death coding in 2005. In Fiji the increase was 6-9 fold which occurred more gradually. Circulatory disease proportional mortality showed inverse patterns that were similar: a sudden 24%–26% decrease in Mauritius in 2005, and a more gradual decrease in Fiji of 33–43% over 2001–2012. The changed CVD and diabetes mortality levels have continued thereafter in each country. These changes exceed by a wide margin the relatively minor changes found in bridge-coding studies from developed countries.

Mortality data are reported routinely by member countries to the WHO mortality database, [10] and cause of death supplied is the underlying cause of death. Underlying cause of death is extracted and coded from the death certification as part of routine mortality processing and vital registration in the source country.

Available information from the Ministry of Health indicates that in Fiji, ICD–10 coding was first introduced in 1999 but was not fully implemented with respect to cause-of-death coding until after 2001. Despite this, the WHO mortality data for Fiji (as supplied to WHO by the Fiji Ministry of Health) are coded according to ICD–9 protocols up to 2000. The more graduated implementation of ICD–10 coding explains the more gradual change in the mortality trends compared to Mauritius. However, lack of reliable mortality data for Fiji in the WHO database from before the introduction of ICD–10 precluded assessment of proportional mortality from this earlier period. Nonetheless, secular trends from 2001 clearly show increasing diabetes and reciprocal decreasing circulatory disease proportional mortality of a broadly similar pattern and magnitude to that of Mauritius.

Examining trends in cause-specific proportional mortality is always fraught because such trends are not independent of changing contributions to overall mortality from other unrelated causes. However, in this instance the magnitude of the increase in proportional mortality from diabetes quite closely matches the magnitude and timing of reduction in cardiovascular proportional mortality. Notable also that during the study period, trends in all-cause mortality rates and life expectancy at birth were stable in Fiji, [12–14] and trends in life expectancy at birth and mortality from non-communicable disease in Mauritius were also stable [15, 16].

These trends contrast with Australia where diabetes and circulatory disease mortality trends prior to and after the introduction of ICD–10 coding in 1998 remained largely unaffected, as demonstrated also in the present analysis, and as documented by the Australian Institute of Health and Welfare report for 1981–2011 [17]. A stable trend of around 20/10^5^ male age-standardised diabetes mortality (as underlying cause), and around 13/10^5^ for females, occurred over 1981–2011. For ‘diabetes associated’ mortality - diabetes in Part I or Part II of the of the death certificate (DC) - rates were 70/10^5^ for males and 45/10^5^ for females. The changes also contrast markedly with the US and European bridge-coding studies, [3, 4] where the highest ICD–9 to ICD–10 related change reported was a 20% proportionate elevation in diabetes mortality in the Netherlands [4]. In other words, changes related to ICD–9 to ICD–10 mortality coding mostly have been minor in the (developed) countries studied.

Considerable variation in placing and sequencing causes of death on DCs in the presence of diabetes has been found. For example, a 1991 Dutch study of 228 physicians, who each compiled DCs from six case histories, found significant variation in assignment of underlying cause of death when diabetes was present [18]. Certifier education and changes in death certification and coding practices were recommended as remedies for misclassifying underlying cause of death. Numerous other studies have focussed on the sources of physician variation in certification practices, including incorrect cause sequencing and multiple causes inserted into single lines [19]. A common finding was of hypertension frequently placed below diabetes in the Part I DC causal sequence, implying that hypertension was a cause of diabetes [20].

Diabetes can be a direct cause of death, and appear in Part I of the DC, when a consequence of a specific diabetes complication covered by ICD–10 codes E10.0–8, E11.0–8, E14.0–8. When diabetes acts as a risk factor, usually along with several others, then it is properly placed in Part II of the death certificate as a significant condition which contributes to death, but does not directly cause it. Atherosclerotic vascular disease can lead directly to death especially through coronary artery disease, including myocardial infarction, and cerebrovascular disease (‘stroke’), and is a consequence of several interacting (modifiable) risk factors, especially high levels of blood pressure and plasma cholesterol (related to obesity, diet, exercise etc.), and tobacco smoking. Diabetes is also a risk factor for atherosclerotic vascular disease, and if cardiac or cerebrovascular disease is the cause of death, then uncomplicated diabetes (if listed) is properly placed in Part II of the DC by the certifier, along with other risk factors for circulatory disease.

The problem appears to be more fundamental than incorrect placement and sequencing of diabetes in DCs. There are no complication categories under diabetes in the ICD–10 code set for coronary heart disease or myocardial infarction, or for cerebrovascular disease or stroke (thrombosis or haemorrhage); these are all in the Circulatory Disease ICD–10 Chapter. Thus ICD coding is congruent with medical understanding, and death certification practice in most countries. However, according to the ICD–10 coding instruction manual, under ‘Accepted sequences’ (Section k, p.58), [21] ‘Acute or terminal circulatory diseases due to other conditions’, is the following instruction: ‘Accept the following acute or terminal circulatory diseases as due to malignant neoplasm, diabetes or asthma:’, and the list of conditions comprises: numerous cardiac and cerebrovascular conditions (Table 1). The mechanical application of this rule necessarily will result in misclassification of circulatory disease death as diabetes as the underlying cause of death when diabetes without (lethal) complications is placed (inappropriately) in Part I of the DC. Notably, the provisions above under ICD–10 instructions, Section k had no counterpart in ICD–9, and are entirely new. Previously under ICD–9, when diabetes ‘not otherwise specified’ or in the absence of a lethal complication (such as renal insufficiency) was in Part I of the DC alongside acute cardiovascular conditions, diabetes may have been ignored or moved to Part II by the medical coders; under ICD–10, the instructions are to retain diabetes in Part I and for it to take priority over other causes excepting cancer or external cause (e.g., death from trauma).

**Table 1:** Extra-diabetes cause-of-death sequences overridden by diabetes as underlying cause of death under ICD–10 coding rules

Condition	ICD–10 code
Acute and subsequent myocardial infarction	I21, I22
Other acute ischaemic heart disease	I24
Pulmonary embolism	I26
Acute pericarditis	I30
Acute and subacute endocarditis	I33
Acute myocarditis	I40
Atrioventricular and left bundle-branch block	I44
Other conduction disorders	I45
Cardiac arrest	I46
Paroxysmal tachycardia	I47
Atrial fibrillation and flutter	I48
Other cardiac arrhythmias	I49
Heart failure	I50
Other ill-defined heart diseases	I51.8
Cerebrovascular diseases	I60–I66,
	I67.6–8,
	I69

In both Mauritius and Fiji, the dramatic changes in diabetes and circulatory disease mortality appear to be related in time to the introduction of ICD version 10, at first abruptly (70–80% of the recorded increase/decrease in the first year), then progressively in each country. This is most likely a consequence of changes in operationalisaton of selection of underlying cause of death under ICD–10 in these countries. It might be argued that an increase in the frequency of uncomplicated diabetes appearing in Part I of death certificates in both Mauritius and Fiji has occurred as a consequence of undoubted rise in diabetes prevalence in both countries accompanying increased blood glucose testing as part of health care, [22–25] especially using portable glucose meters. However, the suddenness and magnitude of the increases in proportional diabetes mortality coinciding with the change to ICD–10 mortality coding renders this explanation implausible for the short term changes, but may contribute to later increasing trends. Without appropriate pre-processing of death certificate data, the introduction of automated mortality coding from DCs (eg, IRIS software[26]) likely will entrench this situation of artefactually raised diabetes mortality and lowered circulatory disease mortality.

From a Mauritius study of mortality outcomes in an adult cohort (aged 20–82 years, *n*=9559) followed from 1987 to 2007 (median follow-up time 15 years), [27] Circulatory disease mortality comprised 54% of total mortality in the cohort, when counting cardiac mortality (38%), cerebrovascular mortality (15%) and death from complications of hypertension (1%) as death from ‘circulatory disease’. Underlying cause of death was coded by the study physicians with veracity of a sub-sample of DCs checked against a random sample (*n*=400) of corresponding hospital records. From the WHO death data, approximately 5% of all mortality in Mauritius comes from those aged < 20 years for 2000–14, and accounting for < 20 year deaths, this cohort estimate of the circulatory disease mortality proportion becomes 52% for all ages, which is close to the proportion for males and females together under ICD–9 mortality coding for this period (Fig. 2).

In Fiji, from cause-specific mortality data reported from a representative cohort study of adults (*n*=2546) followed from 1980 to 1991, [28] we estimate the proportional mortality from diabetes to be 5.9% and 6.3%, males and females respectively for the 11–year period, somewhat higher than proportions for 2001 (cf. Fig. 3) but substantially lower than those reported for 2009–12 (16%–20%, M; 25%–27%, F). Corresponding proportional mortality from circulatory disease is estimated as 52.8% and 49.3% from the 1980-91 cohort study, [28] somewhat lower than for 2001 but considerably higher than over 2009–12 (40%–46%, M; 30%–39%, F).

The findings from these Mauritius and Fiji cohort studies, involving detailed cause-of-death assessment, are more in accord with those from ICD–9 routine coding rather than with those from ICD–10 routine coding.

At face value, the simultaneous rise in diabetes mortality and decline in circulatory disease mortality has attracted no obvious attention nor proffered explanations. These sudden changes cannot be explained by corresponding sudden and opposing nation-level changes in risk factors and their interactions for each mortality outcome. In Fiji, smoking prevalence has declined over the long term, but the decline has not been marked from 2000, [29] and thus changes in smoking prevalence are not a candidate for explaining the apparent sudden drop in circulatory disease mortality in Fiji. Prevalence of hypertension, the other major risk factor for circulatory disease mortality, has increased consistently in Fiji since the 1980s, [30] and thus the increases in this risk factor cannot explain the apparent sudden drop in circulatory disease mortality. Obesity prevalence in Fiji has increased substantially over the last 30 years, with corresponding increases in diabetes prevalence [24]. However, expected mortality outcomes might be increased diabetes mortality and increased circulatory disease mortality (not declines). Trends in prevalence of CVD and diabetes risk factors are not congruent with trends in diabetes and circulatory disease mortality in Fiji.

In the Global Health Estimates Summary Tables for 2011, [9] WHO categorises mortality data from Mauritius as “Reasonably complete death registration data available with underlying cause of death coded using ICD–9 or ICD–10 without excessive use of inappropriate or non-specific codes”; mortality from Fiji is rated as “Data have low completeness and/or issues with cause-of-death assignment which likely affect estimated deaths by cause and time trends” [9]. The examples of Mauritius and Fiji indicate that “excessive use of inappropriate or non-specific codes” is occurring under the aegis of the ICD–10 coding regime.

In Australia and other developed countries, changes in proportional diabetes mortality are not so apparent, but nonetheless are present. A 2010 study by one of the authors (CR) showed the 1999–2006 trend of diabetes proportional mortality (i.e., as underlying cause of death) to increase from 2.3% to 2.7% [31]. Over the same period, there was a minor increase in the proportion of these diabetes deaths comprising diabetes without complications from 74.3% to 77.6%. Further, the proportion of diabetes as underlying CoD, with CVD also in Part I of the DC, increased from 1.6% to 2.1% of all deaths; and the proportion of these diabetes deaths without complication increased from 70.0% to 75.2%. In other words, despite low absolute proportions, around three quarters of diabetes as underlying cause of death arises from ‘diabetes without complications’. This is not isolated to Australia, as the authors found similar results for the US [31]. This situation appears to reflect an increasing trend of medical certifiers to place diabetes in Part I of the DC, combined with ICD–10 coding instructions that prevent removal of ‘uncomplicated diabetes’ from Part I of the DC. In some cases, if ‘uncomplicated diabetes’ is accompanied by chronic renal insufficiency (unspecified) in Part I of the death certificate, an inference may be drawn that the renal disease is a consequence of diabetes. As is evident for Australia and USA, the absolute magnitude of changes in proportional mortality from diabetes and circulatory diseases associated with changes in ICD coding practice were far less than those documented in Mauritius and Fiji.

Errors in placement and sequencing of causes of death at certification could be corrected by experienced manual coders or by pre-processing digitised DCs before application of automated cause-of-death coding software such as IRIS [26]. In many less developed countries, Part II of the death certificate is infrequently used (≤ 5%), and contributory conditions may be placed in Part I of the DC where, according to ICD–10 rules, they are considered as conditions that lead directly to death for the purpose of determination of the underlying cause of death. Thus ‘diabetes without complications’ (E10.9, E11.9, E14.9 in ICD–10) or ‘diabetes, not otherwise specified’, when placed in Part I of the DC will usually become the underlying cause of death, as indicated by the ICD–10 instructions quoted above. The only conditions that override this stipulation are cancer or external cause (injury). Thus coronary heart disease or stroke will not appear as the underlying cause of death when accompanied by uncomplicated diabetes in Part I of the DC since cardiovascular disease is considered as a sequel to diabetes, but diabetes is not a consequence of CVD. However, CVD in diabetics is usually a sequel to other interacting risk factors in addition to diabetes.

A strength of the present study is that it uses whole-population cause-specific mortality data that extends over periods that span changes in cause-of-death coding, and these changes are shown to be quite marked in the case of Fiji and particularly Mauritius. A weakness of the study is that multiple causes of death were not available from the original data assembled from death certificates to enable more detailed analysis at an individual level.

## Conclusions

Large, abrupt changes in diabetes and circulatory disease proportional mortality in Fiji and Mauritius coincided with the local introduction of ICD–10 coding in different years. Diabetes is not nearly as large a contributor to the mortality profiles of Mauritius and Fiji as indicated by official mortality statistics. The progressive replacement of circulatory disease mortality by diabetes mortality implies that cardiovascular disease, the major cause of premature mortality, is decreasing (improving). We have shown that these trends are likely fallacious and bear no plausible relation to trends in population risk factors. These artefacts can undermine accurate monitoring of cause of death for evaluation of effectiveness of prevention and control, especially of circulatory disease mortality which is demonstrably reversible in populations.

Accurate assessment of cardiovascular disease and diabetes mortality is essential for assessing the magnitude of premature mortality at a population level, and of the success or otherwise of actions to prevent and control the diversity of modifiable risk factors and treatment. Action is required to change coding instructions in ICD–10 to prevent such coding artefacts recurring, particularly in developing countries where death certification errors are frequent. International guidelines for recording diabetes in death certification are needed, [32] and recommendations for changes in ICD–10 rules for coding underlying cause of death in the presence of diabetes on the death certificate have been formulated [33]. Mortality data already recorded and based on digitised death certificates with multiple causes of death can be pre-processed to shift ‘diabetes with no lethal diabetes complication’ from Part I to Part II of the death certificate prior to automated re-coding with software such as IRIS. Prospectively, improved information and training is needed for certifying physicians (and medical students) concerning circumstances when diabetes should be placed in Part I or Part II of the death certificate.

## Abbreviations

CVD: Cardiovascular disease
DC: Death certificate
F: Female
ICD: International classification of diseases
IHME GBD: Institute for health metrics and evaluation global burden of disease
M: Male
T2DM: type 2 diabetes
WHO: World health organization
YLL: Years of life lost
